# Tannin Poisoning

**Published:** 1892-02

**Authors:** 


Tannin Poisoning.—The fauces of a young gentleman were flushed over with a solution of Tannin 1.15, which was immediately followed by great swelling of the mucous membrane, obstruction of the nose, and copious watery secretion, great œdema of the uvula and of the soft palate. An hour later dullness of the mind and unbearable itching followed by an universal urticaria.—*Translated by S. L.*

